# History and Evolution of the Electroencephalogram

**DOI:** 10.7759/cureus.66385

**Published:** 2024-08-07

**Authors:** Alyssa Arjoonsingh, Benjamin C Jamal, Latha Ganti

**Affiliations:** 1 Biomedical Sciences, University of Central Florida, Orlando, USA; 2 Biomedical Engineering, Brown University, Providence, USA; 3 Emergency Medicine & Neurology, University of Central Florida, Orlando, USA; 4 Research, Orlando College of Osteopathic Medicine, Winter Garden, USA; 5 Medical Science, The Warren Alpert Medical School of Brown University, Providence, USA

**Keywords:** tools in medicine, medical education, history of medicine, eeg, electroencephalogram

## Abstract

This paper summarizes the history and evolution of the electroencephalogram (EEG). The EEG, used to record the electrical activity of the brain, is a pivotal tool in neuroscience and medicine. Its history and evolution reflect significant advancements in our understanding of brain function and our ability to diagnose and treat neurological conditions. This tool has revolutionized our understanding of the brain's electrical activity and is the cornerstone for the diagnosis and treatment of epilepsy and related disorders. The evolution of the EEG from early experimental observations to sophisticated modern applications highlights the profound progress in our ability to monitor and interpret brain activity. The EEG remains an invaluable tool in clinical and research settings, continually evolving with technological advancements to expand our understanding of the human brain. This review traces the journey of this iconic tool.

## Introduction and background

An electroencephalograph (EEG) is a vital instrument used in medicine, mainly in the neurologic field, with its prominent use being to measure electrical activity in different areas of the brain. After several decades of modification and advancement, EEG uses electrodes and coin-sized metal disks connected to thin wires [1]. An EEG recording displays electrical impulses sent out through brain cells to communicate and identifies obscure frequencies or patterns in the brain's activity. Obtaining information with an EEG allows physicians to distinguish and analyze disorders manifested in the brain, such as epilepsy, sleeping disorders, brain tumors, head injuries, behavioral delays, disorders, infections in the brain, or even geriatric conditions such as Alzheimer's, and much more. However, the use of an EEG has not always been accessible.

Richard Caton discovered electrical activity in organisms, specifically rabbits and monkeys. Caton used a galvanometer to measure electrical activity in the cortex of these animals, and Emil du Bois Reymond, Hermann Helmholtz, and Julius Bernstein later continued the work on electrical activity in organisms. Bernstein pioneered the first device to measure the velocity of biological electric impulses, called the differential rheotome, in 1868. The differential rheotome would analyze the action potential and transmission of electrical impulses to the nervous system [2].

Similarly, Angelo Mosso pioneered early neurological imaging by measuring cerebral blood flow during mental activities. He utilized brain pulsations detected through skull breaches, representing a primitive precursor to modern human neuroimaging techniques. Regarding the development of electrical impulse evaluation in the brain, Hans Berger is known for the first EEG and human record of biological electricity during neurosurgical operations. His findings were coined "alpha and beta" waves, and he noticed they would alter due to different physical and mental effort or even cerebral injury on the brain. Following Hans Berger's invention of the EEG, Herbert H. Jasper would go on to study the state of consciousness, developmental learning, epilepsy, and monitoring the activity of a single neuron [3]. Jasper evolved the EEG by identifying the proper electrode placement for signal processing techniques. William Grey Walter further advanced EEG readings by refining methods to interpret different rhythms, such as the frequencies of alpha and beta waves and what they indicate in the state of cognitive processes. Walter discovered how to identify brain tumors in parts of the brain using delta waves, which slowed activity [4] and paved the way for brain-computer interfaces with his invention of the Machina Speculatrix. The Machina Speculatrix helps with identifying functional disabilities and communication deficits by using electrical impulses recorded from the EEG to interact with external objects [5].

As we now have more advancements in medical machinery, such as magnetic resonance imaging, computed tomography scans, and cerebral arteriograms, the implications and discoveries found using an EEG are significant. The importance of its evolution is critical to understanding its vital role in healthcare as well as potential advancements to enhance its performance.

## Review

**Figure 1:**
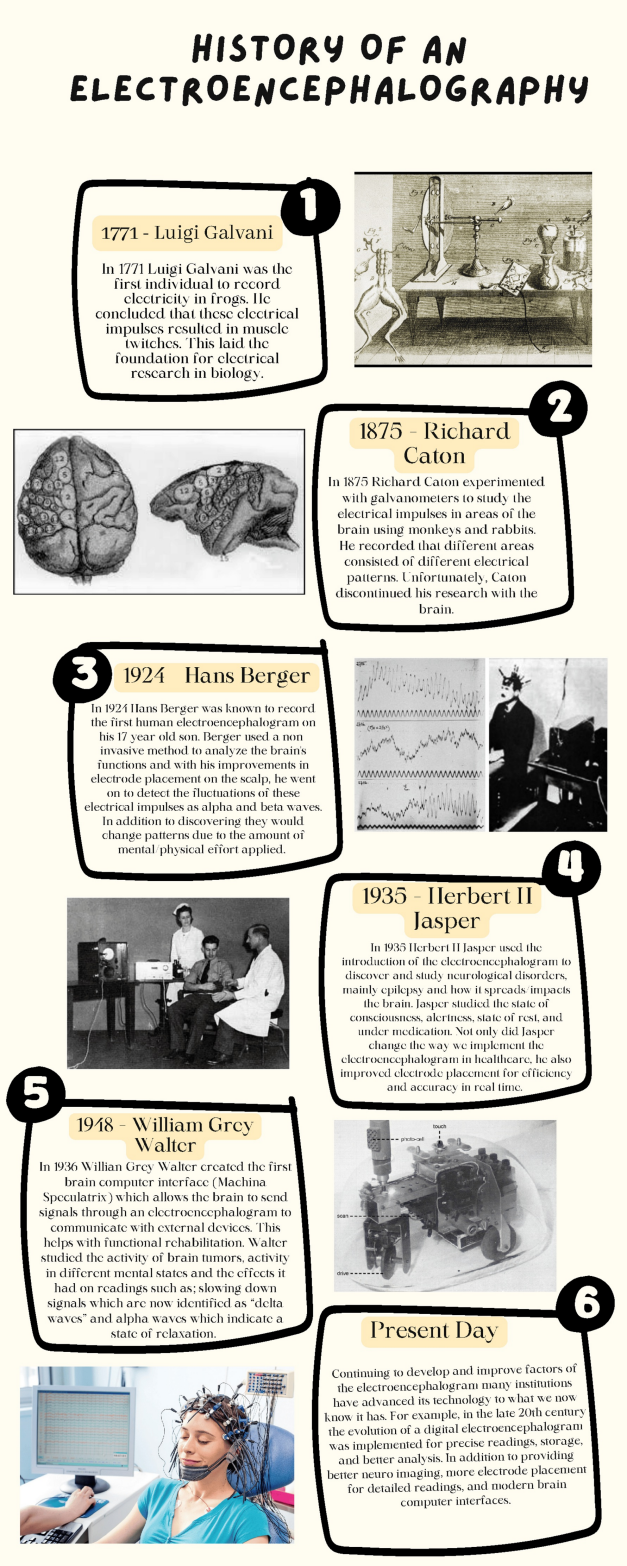
Infographic depicting the history of the electroencephalogram. Designed by Alyssa Arjoonsingh on Canva.com. All images in the infographic are from Creative Commons sources.

Figure 1 displays numerous early pioneers who contributed to the development of the EEG.

The majority of these initial discoveries were primarily conducted on animal models. The earliest known examples are from Luigi Galvani and Richard Caton. In the late 18th century, Galvani experimented with electrical currents on frog legs. Galvani observed the activity of muscle twitches and its reaction to such current, concluding there must be a reaction to the action [6]. The reaction, which was muscle twitching as a result of electrical impulses, laid the foundation for research on biological electrical activity. Richard Caton later built on this in the 19th century using a galvanometer, originally designed to measure the direction and velocity of current in a circuit on the exposed cerebral cortex of monkeys, cats, and rabbits. With this technique, it was brought to his attention that current was detected within the cortex in small frequencies, as the galvanometer was not designed to detect such signals. More experimentation came with more discoveries as the fluctuation of brain waves occurred due to stimulation to muscle movement or change in behavior. He stated in his research that "shining a bright light on the retina led to variations in currents in the posterior and lateral parts of the brain" [7]. Caton could be known as the first individual to record electrical activity in an organism's cerebral cortex and document changes in current from stimulation; many others played a pivotal role in expanding his discovery.

Angelo Mosso began neuroimaging in humans in 1880. Mosso used his profound method of human circulation balance to assess "the redistribution of blood during emotional and intellectual activity" [8]. Not only did this lay the foundation for non-invasive neuroimaging, but it also indicated that blood volume changes by brain pulsations. Hans Berger first successfully completed assessing electrical impulses in the brain using non-invasive neuroimaging on a human for the first time in 1924. During a neurosurgical operation, Berger created the first EEG using a more sensitive double-coiled galvanometer on his 17-year-old son [9]. He took three official recordings of electrical activity in the brain and discovered how to use them to observe disorders fostered in the brain. The first of his observations was the string galvanometer, which demonstrated how brain waves slow down approximately 6-8 seconds in patients with cerebral injury. The second is his direct recordings from the cortex and white matter, proving the presence of neural and complex sensory functions on an EEG. The third highlights his recordings of complex brain waves resulting in epileptic and absence seizures. The two most prominent waves he viewed were the alpha and beta. After discovering the alpha wave, he observed another medium wave with lower amplitude and higher frequencies, naming it the beta wave [10]. Berger observed changes in recordings due to cerebral injury, attention, and mental effort and implemented the creation and use of the first EEG to identify such abnormalities in patients.

Many other neurologists and physicians have advanced the EEG to what we know today, but the main contributors are Herbert H. Jasper and William Grey Walter. Herbert H. Jasper was significant to the development of the EEG by creating microelectrodes to monitor a singular neuron [3]. Jasper developed the 10-20 electrode placement system, ensuring all brain areas are covered for efficiency in EEG readings. The 10-20 placement system covers the frontal, parietal, temporal, and occipital areas [10]. In 1932, Jasper studied the phenomenon of fluctuating alpha waves being slowed down approximately 2-3 seconds and concluded they could result from brain damage, identifying them as delta waves [3]. Jasper then furthered his research by localizing areas of the brain prone to epileptic seizures by analyzing the disturbance of brain waves. He implemented the EEG during neurosurgery and in clinical settings and discovered how to use these readings to enable diagnosis and treatment for neurological disorders, mainly epilepsy.

William Grey Walter continued the research of delta waves initiated by Herbert H. Jasper, which was most significant for integrating the study of the brain using the EEG and brain-computer interfaces. Walter concluded that the reduction of delta brain waves can be a result of a brain tumor, in addition to noting that alpha waves reflect a state of relaxation in the brain. He emphasized the importance of theta waves in identifying lesions and how we can use the EEG to define sub-cortical tumors using special depth-constructed electrodes [4]. Walter was a co-inventor of the frequency analyzer and the EEG toposcope, both of which created a new path for analysis. The frequency analyzer "gives an on-line analysis of the frequencies that are made of the electroencephalography trace every 10-second period" and the toposcope allowed physicians to "draw a snapshot map projecting the electrical data visually on a spatial coordinate system that can be laid out to represent a simple model of the head" [4]. Both of these were implemented in the clinical setting. Walter highlighted that an EEG can be used to identify abnormal changes in behavior and used to study psychiatry. To demonstrate this, he created a brain-computer interface called the Machina Speculatrix to visually identify complex and unpredictable behavioral habits through brain signals [4]. It was a vivid demonstration of the function of the human brain when it undergoes specific disorders and pressure. The Machina Speculatrix has advanced to present-day microchips implanted in the brain to control prosthetics.

By the mid-20th century, the EEG had become a standard tool in neurology for diagnosing epilepsy, sleep disorders, encephalopathies, and brain death. The development of multi-channel EEG allowed for a more detailed mapping of brain activity. The advent of computers further revolutionized EEG. Digital signal processing enabled more sophisticated analysis, such as Fourier transformation, to decompose complex EEG signals into their constituent frequencies. Subsequently, the development of quantitative EEG emerged. This technology records digital EEG signals, which are input into complex mathematical algorithms for analysis. Quantitative EEG allows for analysis of specific frequency band and signal complexity, connectivity, and network analysis [11]. Towards the later part of the 20th century, high-density EEG emerged. These systems have 128 or 256 electrodes, providing high-resolution spatial mapping of brain activity. Next, advances in technology led to the creation of portable and wearable EEG devices, broadening the applications of EEG in everyday settings, sports, and sleep studies. Modern EEG analysis increasingly incorporates machine learning and artificial intelligence to improve the accuracy of diagnoses and to uncover new patterns in brain activity data [12].

## Conclusions

The development of the use and structure of the EEG is critical to the neurologic field and understanding of the brain. The EEG is ever-changing for efficiency, whether it be the different principles of the brain and methods used to analyze different disorders, areas, or even activity. The evolution of the EEG began with identifying the presence of electrical activity in frog legs and has now evolved to identify epilepsy, dementia, brain injuries, psychosis, insomnia, stroke, tumors, and more. In modern medicine, several types of EEGs exist, such as prolonged, ambulatory, video, routine, and sleep. As the EEG continues to evolve, we hope to see better efficiency, accuracy, and advancements in its potential.
